# Supplementary material for: CRISPR/Cas9 screening using unique molecular identifiers
Source: Mol Syst Biol. 2017 Oct 9;13(10):945. doi: 10.15252/msb.20177834 (PMC5658704; doi:10.15252/msb.20177834)
Supplement: Supplementary file 1 — Expanded View Figures PDF [file MSB-13-945-s001.pdf]

Expanded View Figures

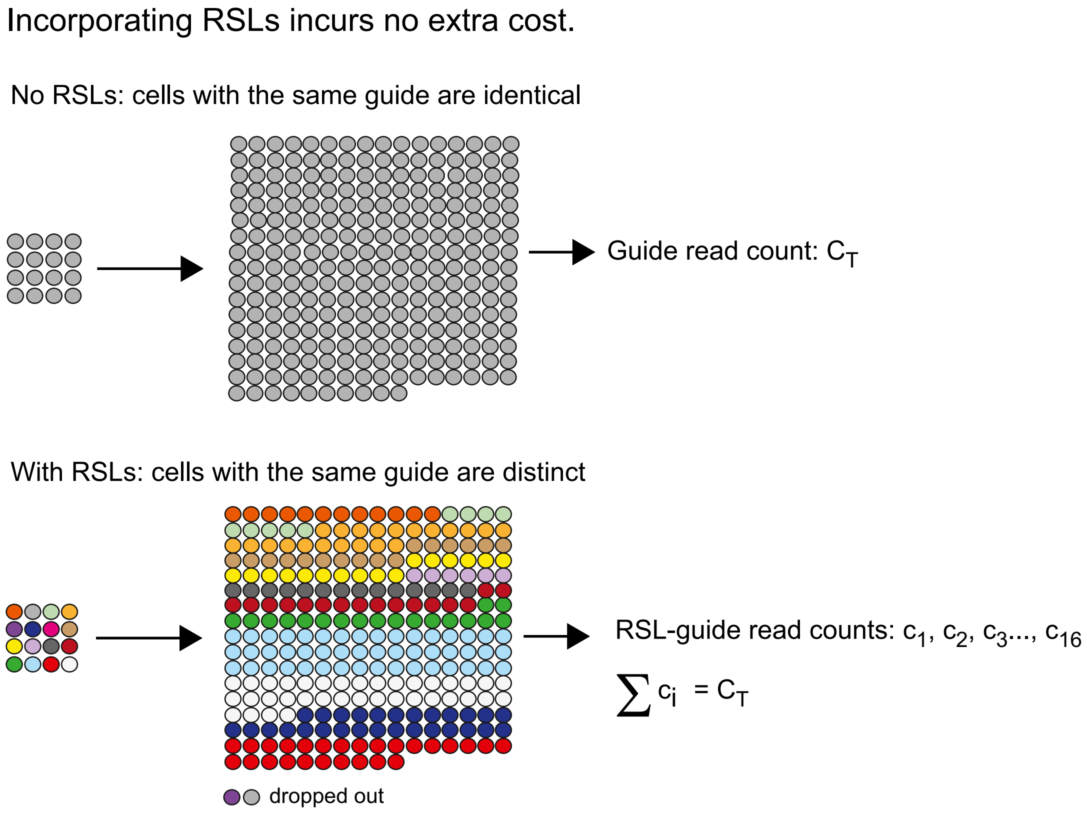

Figure EV1. Incorporation of RSLs incurs no extra cost. Example cartoon of the absence and the presence of RSLs.

Top: In the absence of RSLs (total count analysis), the progeny derived from distinct cells transduced with the same guide are indistinguishable from one another. Sixteen cells harboring the same guide are shown proliferating for several generations. The only readout obtained is a single value, the read count of the common guide (total count, C_T). Bottom: In the presence of RSLs, the progeny derived from distinct cells transduced with the same guide become distinguishable (distinctly colored cell lineages). Lineage heterogeneity becomes apparent, and a distribution rather than a single value is obtained. Lineage dropout becomes available as an additional binary and thus robust readout. Importantly, as illustrated here, the inclusion of RSLs does not require scaling up of existing protocols, the number of cells per guide can remain the same. The lower read counts obtained per RSL-guide (c_i) sum up to the total readcount for the guide.

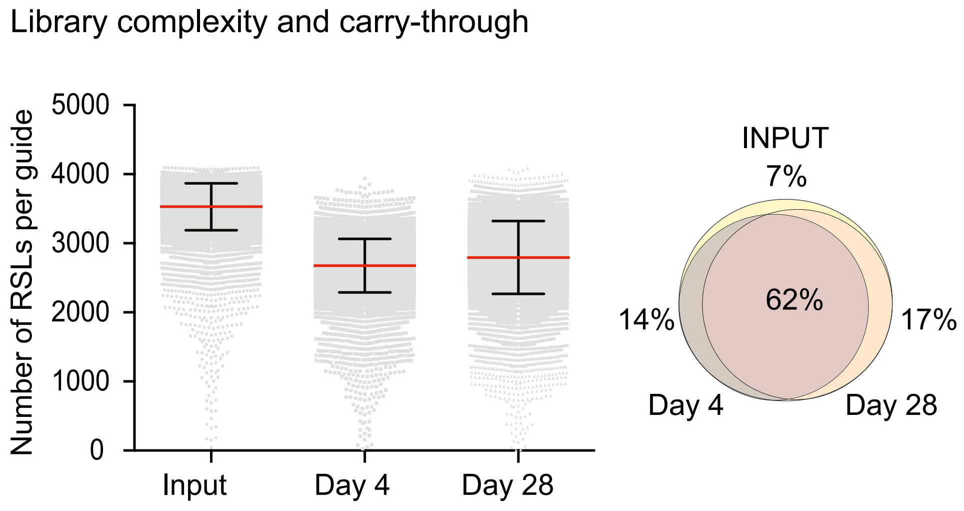

Figure EV2. Number of distinct sequences carried through the experiment in one representative experimental replicate.

Left: Boxplot. An average of 3,600 RSLs per guide in the plasmid library reduced to an average of 2,800 RSLs per guide in the samples taken from the cell populations. Many of these RSLs have very low read counts, those were filtered out and not used for downstream data analysis. Red line, average. Error Bars, standard deviation. Right: Venn diagram. Both time points together covered 93% of input RSL-guides (78 million unique sequences in the cell population). The overlap between Day 4 and Day 28 was two-thirds, with about one-sixth of sequences found either only in Day 4 or only in Day 28. This is a consequence of unavoidable undersampling, which also occurs in the absence of RSLs, however in their presence becomes apparent and allows removal of inconsistently sampled lineages.

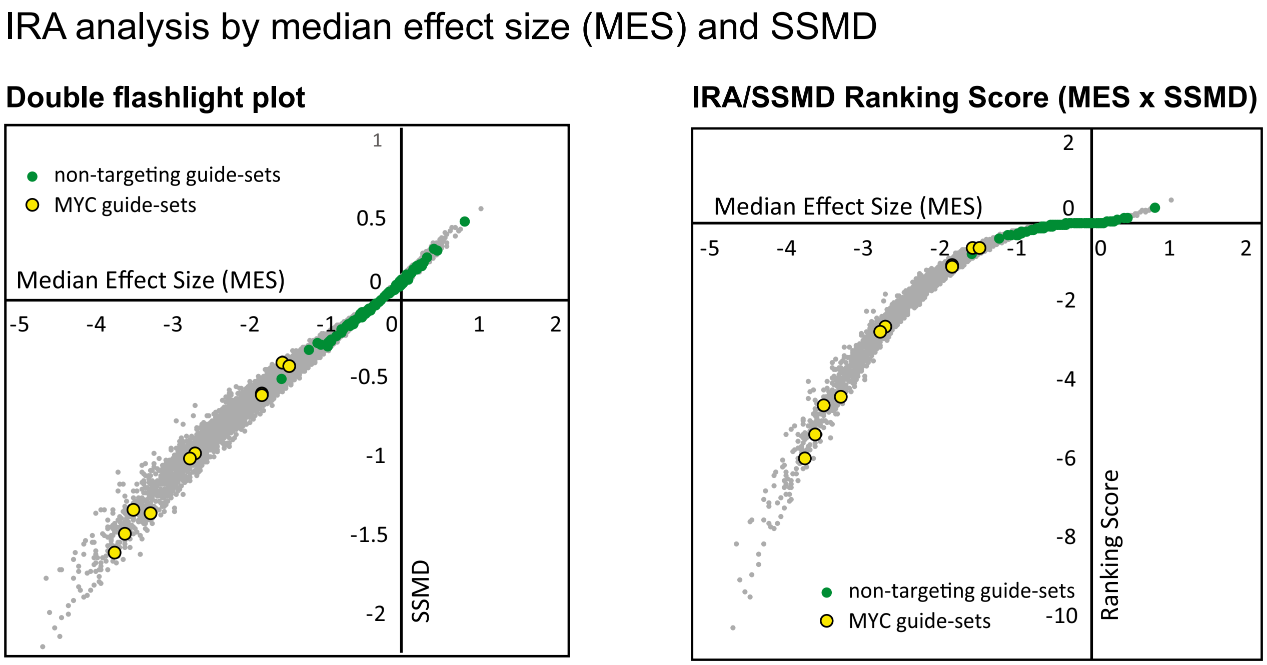

Figure EV3. Internal replicate analysis by strictly standardized mean difference (IRA/SSMD).

Left: Double flashlight plot. SSMDs for all >23,000 guide-sets are plotted against their median effect sizes (MES) for 64 internal replicates. Green circles indicate non-targeting control guide-sets, and yellow circles indicate the 10 guide-sets targeting MYC. Right: Ranking Score. A hit score was defined as the product of MES and SSMD. This score is negative for depleted guides and positive for enriched guides, and was used for guide ranking. The score was plotted against the median effect size for all guides. Green circles indicate non-targeting control guide-sets, and yellow circles indicate the 10 guide-sets targeting MYC.

© 2017 The Authors

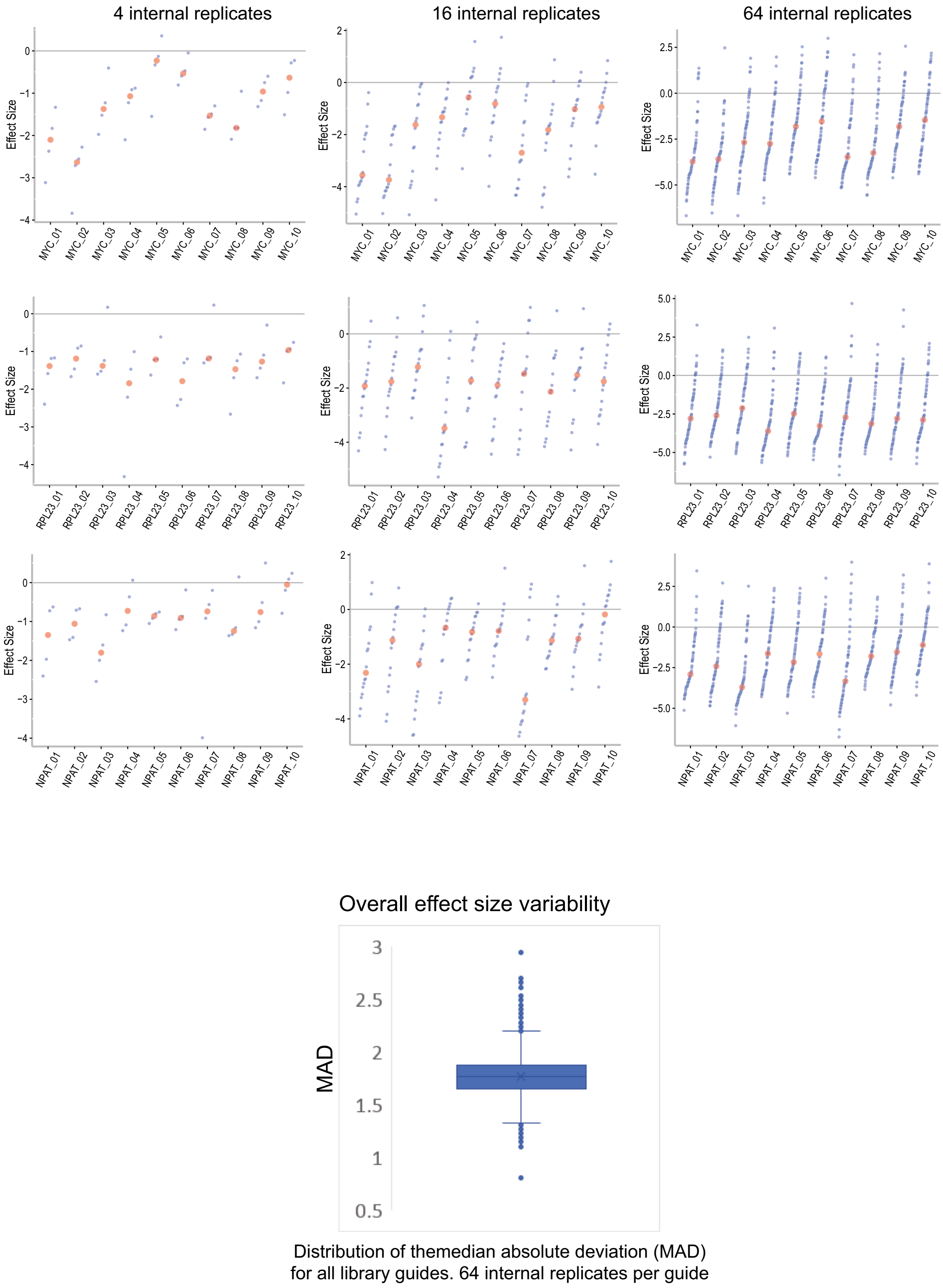

Figure EV4. Variability of internal replicates.

Top: As in Fig 2A. Additional examples of variability in effect size after binning into different numbers of internal replicates (4 internal replicates, left; 16 internal replicates, center; 64 internal replicates, right). Bottom: Boxplot showing the distribution of the variability across the whole data set for 64 internal replicates. The median absolute deviation in effect size (log2 fold change) is plotted for all guides in the library. Note that the spread is substantial, this is the case whether or not RSLs are present, but can only be detected in the presence of RSLs. Box, first and third quartiles; whiskers, 1.5-fold interquartile range; line, median; cross, average. Outliers are shown.

© 2017 The Authors

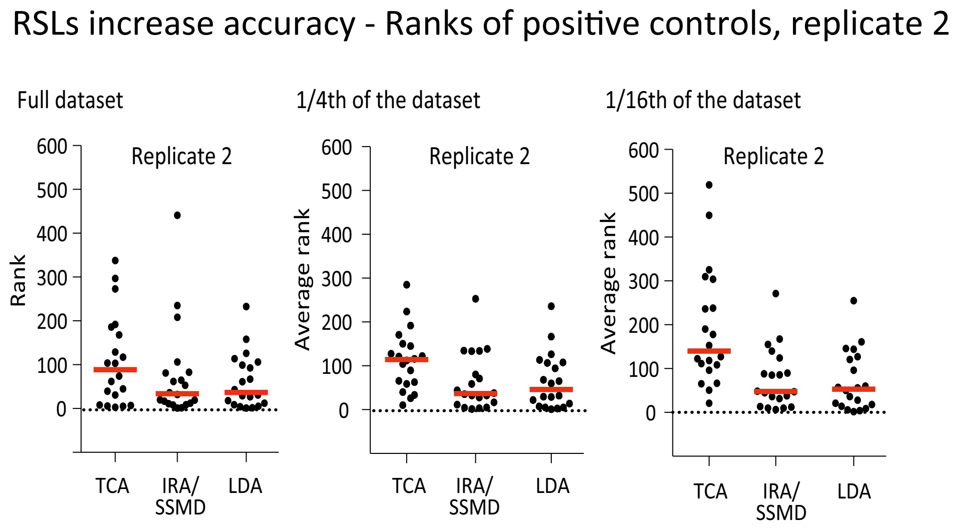

Figure EV5. RSLs increase accuracy of hit calling.

As in Fig 3A, but for the second experimental replicate.

© 2017 The Authors
